# Determination of physical properties of undisturbed soil samples according to V. Novák

**DOI:** 10.1016/j.mex.2023.102133

**Published:** 2023-03-12

**Authors:** Marko Spasić, Oldřich Vacek, Kateřina Vejvodová, Václav Tejnecký, Filip Polák, Luboš Borůvka, Ondřej Drábek

**Affiliations:** a Department of Soil Science and Soil Protection, Faculty of Agrobiology, Food and Natural Resources, Czech University of Life Sciences Prague, Kamýcká 129, Prague 6, Suchdol, Czech Republic; b Department of Landscape Architecture, Faculty of Agrobiology, Food and Natural Resources, Czech University of Life Sciences Prague, Kamýcká 129, Prague 6, Suchdol, Czech Republic

**Keywords:** Cylinder, Kopecký, Physical soil properties, Bulk density, Particle density, Porosity, Water retention, Saturation, Gravimetry, Pycnometer, Determination of physical properties of undisturbed soil samples according to V. Novák

## Abstract

The methodology described here presents the procedures for determining physical soil properties of undisturbed soil samples. Besides describing the methods for determining bulk and particle density, moisture content and porosity of the soil in detail, it also offers a way of determining soil's water holding properties when there is no pressure membrane apparatus available. This method is based on a capillary water saturation experiment and gravimetric measurements performed in different time intervals after the saturation (30 minutes, 2 hours, and 24 hours). With a few, simple to follow steps, and not using complicated and space-consuming equipment, it can be replicated in almost any laboratory, and the results are easily interpreted. The method was, and still is, widely used in the Czech Republic, and some parts of it are used as standard soil testing methods. To a lesser or greater detail, this method is described in Rejšek (1999), Valla et al. (2011), Pospíšilová et al. (2016) and ÚKZÚZ (2016), and this methodology is compiled from those publications, mainly focusing (and using the same abbreviations) on the procedures described by Valla et al. (2011). The methodology described does not essentially differ from the original, but the steps here have been described to a greater detail, based on the practical experiences obtained over the years, in order to make some common mistakes less likely to happen. The methodology is further complemented with graphical illustrations for each step described in the process, making it clearer, more easily understood, and easier to replicate. Since this methodology has not been available in English so far, this guide offers a great opportunity of its replication on an international level.

• Simple, cost-effective and environmentally friendly method for determining physical soil properties
• Easy replication and results interpretation
• Results can be obtained even in non-highly specialized soil laboratories

## Graphical abstract

**Figure:**
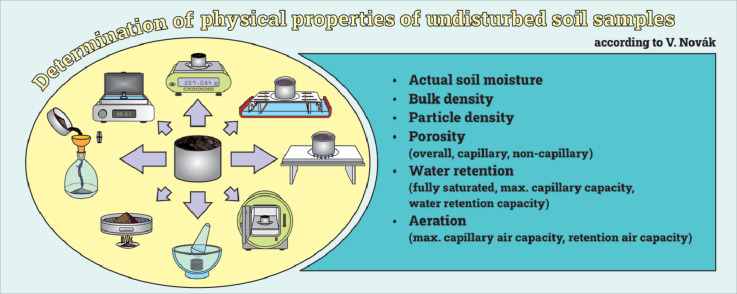

Specifications table

**Table:** 

Subject area:	Environmental Science
More specific subject area:	Soil Science
Name of your method:	Determination of physical properties of undisturbed soil samples according to V. Novák
Name and reference of original method:	Valla, M., Kozák, J., Němeček, J., Matula, S., Borůvka, L., & Drábek, O. [15]. Pedologické praktikum (2nd ed.). Česká zemědělská univerzita v Praze. ISBN 978-80-213-0914-2 p.p. 28-32 (in Czech)
Resource availability:	https://www.royaleijkelkamp.com/products/augers-samplers/soil-augers-samplers/undisturbed-core-samplers/soil-sampling-ring-kit-model-c53/ https://www.kavalier.cz/en/bottle-specific-gravity-gay-lussac-sp142.html

## Introduction

Undisturbed soil samples are used for determination of soil's basic physical properties. The samples are usually taken by using steel cylinders which have a constant volume. One of the main results that can be obtained from the undisturbed soil sample is the dry bulk density – the ratio of mass and volume of the dry soil sample with pores and caverns. Bulk density of samples with different moisture contents, as well as different compaction levels, can be determined by using the same principle. Saturating undisturbed samples with water and observing the way water drains gravitationally or under certain pressures can provide us with valuable information about soils’ water holding properties. By disturbing an undisturbed soil sample, various other physical and chemical soil properties can be determined. Particle density – the ratio of mass of solid soil fractions and the volume of those solid fractions (without pore space between them) is determined from disturbed soil samples. Combined, all of these results are essential in different branches of science and engineering – environmental sciences, agricultural and forestry sciences, civil, mining and materials engineering, etc. Directly, bulk density plays an important role in many engineering calculations (calculation of forces that affect slope stability, bearing capacity, compaction, etc.). Indirectly, with other data, it can be used for assessment of storage capacity of various nutrients or elements of a certain area (e.g. carbon storage), or, combined with particle density, it is used for calculation of soil's porosity – the amount of pore space in the soil. Through porosity, an assessment of soil compaction level can also be obtained (this is mostly used in agricultural calculations, whereas other methods for achieving and measuring various compaction levels, like the Proctor test, penetrometer tests and plate tests are more commonly used in engineering and geomechanics).

Amount of water present in the soil (determined on undisturbed soil samples) under different conditions is, again, very important parameter for both engineering, agriculture, forestry and environmental sciences. In engineering and geomechanics, the amount of water in the soil can not only drastically change the forces applied, but also change the calculation formulas depending on the condition the soil is in (for example – naturally wet, submerged, or fully saturated soil). From the perspective of agriculture, forestry and environmental sciences, soil air and water holding properties are very important physical soil parameters which give insight into the amounts of plant-acessible water in different soils, and the relations between air and water in various pores, which are often used as some of the indicators of soil fertility.

The method described here has been used for processing undisturbed soil samples in the Czech Republic for a very long time, since it represents a basic method for determination of bulk and particle density, porosity and moisture content that's used all over the world and is a part of many national and international standards, and besides that, provides a simple and low-cost method for determination of soil water retention parameters, which can be obtained in almost any laboratory. This method has already been used in a multitude of researches, including journal publications and conference proceedings [2,6,14], BSc., MSc. and doctoral dissertation theses [1,3,4,12], study practicums and scripts [5,9,15], laboratory standards, books and monographs [8,11,13,16]. Many Ministry and Government issued documents in the Czech Republic consider the parameters obtained from this method to be one of the crucial factors for the assessment of overall soil quality in agriculture, forestry and environmental sciences [7,10]. Although being very commonly used on aregional level due to the reasons mentioned above, the lack of materials available in English made it difficult to cite and reproduce on an international level.

## Method details

Undisturbed soil samples are used for determination of soil's basic physical properties. Samples are taken in Kopecký steel cylinders (V_S_=100 cm^3^). Before the sampling, each cylinder (which has a specific marking) is weighed (empty and without caps), and its mass (G_V_) is recorded. Undisturbed soil samples (according to V. Novák) are used to determine the actual humidity (of naturally wet soil), capillary saturation, maximum capillary capacity, overall porosity and maximum water capacity. All of the procedures described here are performed on a single Kopecky cylinder sample.

### Materials needed

(a) Kopecky steel cylinder (V=100 cm^3^) with a sample
(b) Laboratory scale (up to 1 kg, min. precision 0.01 g)
(c) Analytical scale (precision 0.001 g)
(d) Pycnometer (Gay-Lussac, 100 ml) with a funnel
(e) Water bath
(f) Laboratory oven (at 105°C)
(g) Sieve (mesh diameter 2.00 mm)
(h) Mortar and pestle
(i) Watch glasses (2, one for weighing the sample and one for preventing evaporation)
(j) Filter papers (small-circular and large-square)
(k) Plastic tray and stand for water saturation
(l) Beaker or a metal bowl
(m) Wash bottle with distilled water

### Laboratory procedures

**Fig. 1:**
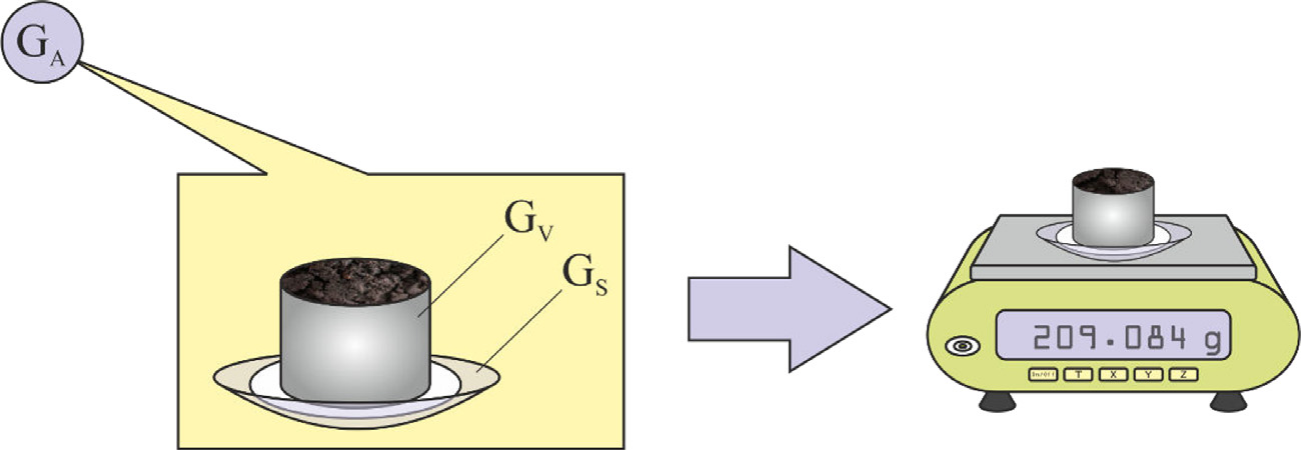
Step 1: weighing of the fresh (naturally wet) soil sample.
(2) The sample is saturated with distilled water by capillary rise through a filter paper placed on a perforated stand, where the sides of the filter paper are dipped in water. The upper side of the cylinder is covered by a watch glass, in order to prevent evaporation (Fig. 2). The sample is saturated for 24 hours, or until the upper surface of the sample becomes wet and shiny.

(1) As soon as the sample is brought to the lab, the cylinder with fresh soil sample (with the caps taken off) is weighed on laboratory scales (G_A_), with the bottom side positioned on a circular piece of filter paper, placed on a watch glass of known mass (G_S_). This step is shown in Fig. 1.

**Fig. 2:**
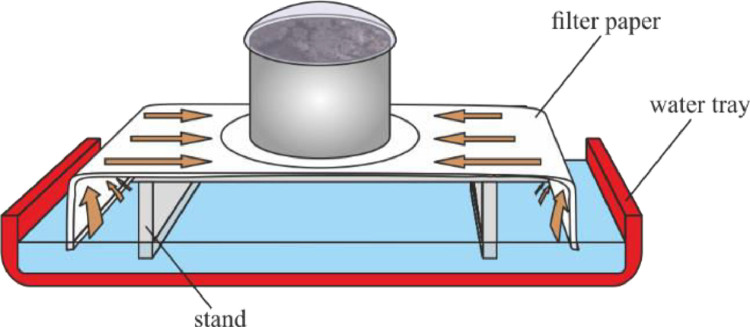
Saturating the sample with water through a filter paper.
(3) The sample with its filter paper is moved to the side, so the excessive water can drip out of the filter paper. It is put on the watch glass that's already been used for weighing (G_S_) and its mass (G_B_) is recorded (Fig. 3).

**Fig. 3:**
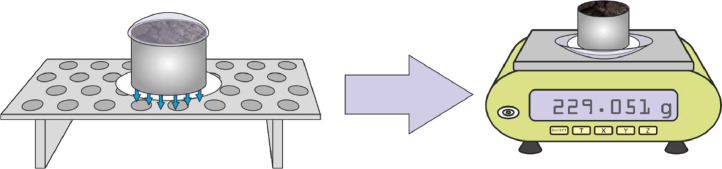
Measurement of a fully saturated sample.
(4) The sample is put on a 4 times folded filter paper, and the upper side is covered by a watch glass. This is the hour zero, and the time of desaturation is calculated from this point (Fig. 4).

**Fig. 4:**
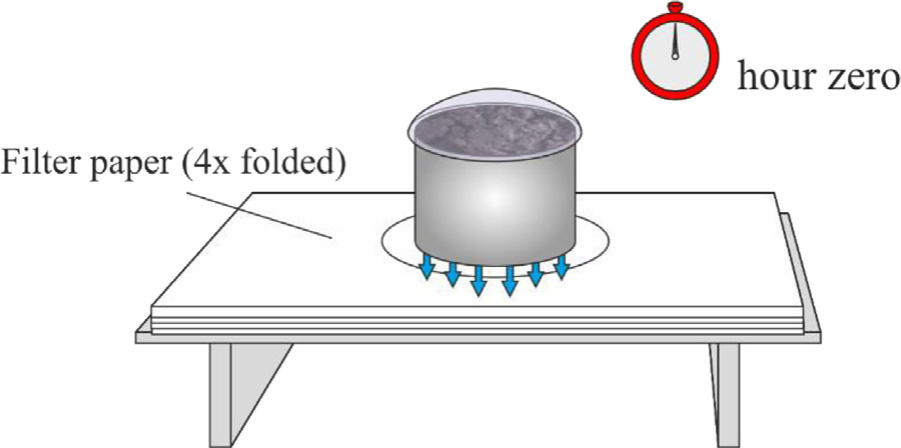
Hour zero, the moment desaturation time is measured from.
(5) The cylinder, covered by a watch glass, is drained for 30 minutes, and is weighed (G_C_) on the watch glass (G_S_) already used for the previous measurement.
(6) The cylinder, covered by a watch glass, is again set on a piece of 4 times folded, dry filter paper, and is weighed on the watch glass (G_S_) after 90 minutes (2 hours from hour zero) (G_D_).
(7) Once again, the sample, covered by a watch glass, is set on a 4 times folded, dry filter paper, and weighed with the previously used watch glass (G_S_) after 22 hours (24 hours from hour zero) (G_E_). Steps 5, 6 and 7 are graphically represented in Fig. 5.

**Fig. 5:**
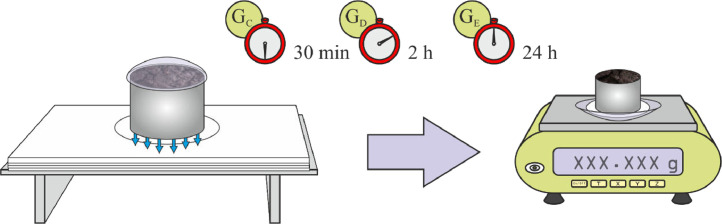
Weighing the sample 30 minutes (G_C_), 2 hours (G_D_) and 24 hours (G_E_) from hour zero.
(8) The cylinder, with the circular filter paper and the watch glass (G_S_) is dried in an oven at 105°C until a constant mass is reached, and weighed (G_F_) (Fig. 6).

**Fig. 6:**
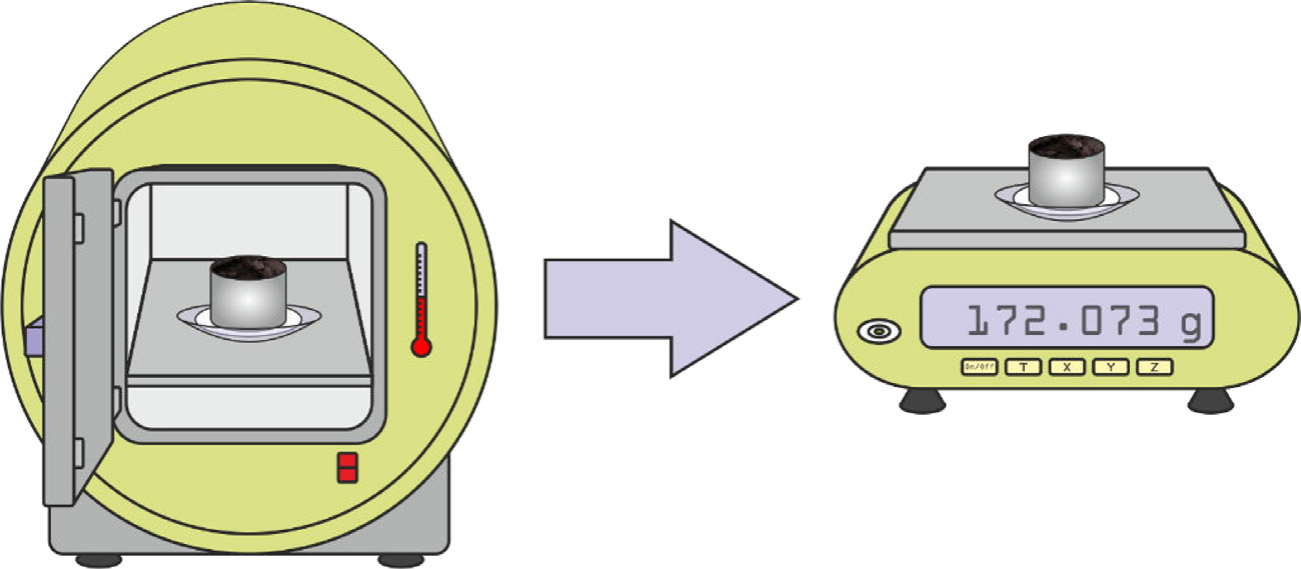
Sample drying in the oven at 105°C until constant mass (G_F_).
(9) The dried sample can then be removed from the cylinder, crushed, sieved through a 2.00 mm sieve and prepared for the pycnometer method and determination of particle density (Fig. 7).

**Fig. 7:**
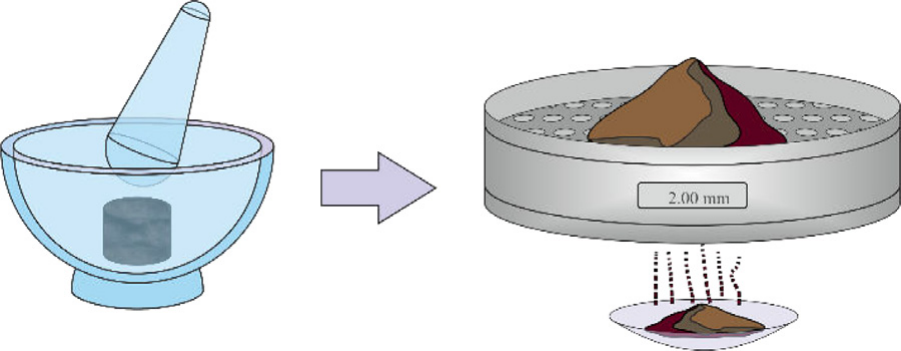
Sample crushing and sieving.
  A pycnometer, filled with distilled water, is tempered to 20 ± 0.5°C in a water bath (without the cap) for 30 minutes and weighed (P_H2O_) after the cap has been added (the entire capillary in the cap needs to be filled with water) and the outside of the pycnometer is dried with a towel (Fig. 8).

**Fig. 8:**
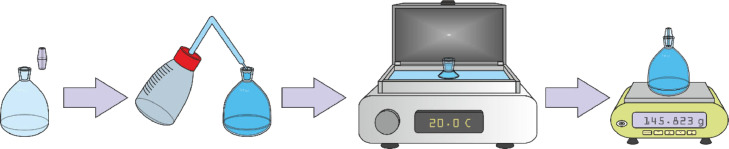
Pycnometer with water preparation and measurement.
  Approximately 10 g of soil (n) is weighed into a beaker (or a metal bowl) using an analytical scale (the exact value used needs to be recorded), and boiled in a small amount of distilled water for 5 minutes. The water is removed from the pycnometer, and the boiled soil suspension is carefully transferred to the pycnometer. It is essential to ensure that all of the soil is transferred to the pycnometer (for this purpose, a wash bottle is used to wash the soil from the beaker and the funnel). The pycnometer is then carefully filled up to the top with distilled water and tempered in the water bath for 30 min (without the cap). The pycnometer is weighed (P_Z_) after the cap is installed and the outside of the pycnometer and the cap is dry (Fig. 9). The final particle density value (ρ_z_) is calculated as the average value obtained from 2 measurements, where the difference between the samples should not be greater than 0,03 g/cm^3^.

**Fig. 9:**
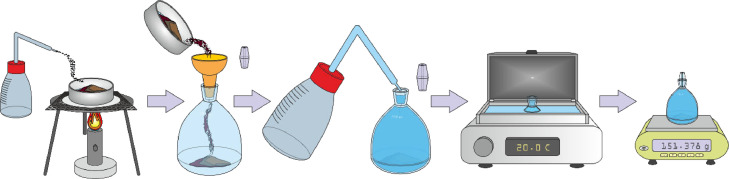
Soil suspension preparation and measurement.

All the measurements, symbols, formulas and units that derive from this methodology are presented in Table 1

**Table 1:** Calculations.

Measurement	Symbol	Calculations	Unit
Actual moisture	θ_mom_	(G_A_-G_F_)*	% (volume)
Saturation	θ_ns_	(G_B_-G_F_)*	
30 minute moisture	θ_30_	(G_C_-G_F_)*	
Max. cap. water capacity	θ_MKK_	(G_D_-G_F_)*	
Water retention capacity	θ_RVK_	(G_E_-G_F_)*	
Particle (specific) density	ρ_z_	n/(n+P_H2O_-P_Z_)	g/cm^3^
Dry sample mass	G_H_	G_F_-(G_V_+G_S_)	g
Bulk density	ρ_d_	G_H_/V_S_	g/cm^3^
Porosity	P	((ρ_z_-ρ_d_)/ρ_z_)*100	% (volume)
Capillary porosity	P_k_	θ_RVK_	
Non-capillary porosity	P_n_	P-θ_30_	
Aeration	V_z_	P-θ_mom_	
Max. capillary air capacity	K_MKKvz_	P-θ_MKK_	
Retention air capacity	K_RVKvz_	P-θ_RVK_	

⁎ in cases where the Kopecky steel cylinder volume is not 100 cm^3^, the resulting value should be recalculated with the cylinder volume that was used. G_A_ – mass of naturally wet soil sample [g] G_B_ – mass of fully (capillary) saturated sample [g] G_D_ – sample mass 30 minutes after hour zero [g] G_D_ – sample mass 2 hours after hour zero [g] G_E_ – sample mass 24 hours after hour zero [g] G_F_ – sample mass after drying at 105°C [g] G_H_ – „pure“ (soil only) sample mass after drying at 105°C [g] G_V_ – mass of the steel cylinder [g] G_S_ – mass of the watch glass [g] n – mass of soil used for the pycnometer method [g] P_H2O_ – mass of pycnometer with distilled water [g] P_Z_ – mass of pycnometer with the soil suspension [g] V_S_ – cylinder volume [cm^3^]

Bulk density (ρ_d_) of the soil is defined as the density of soil with pores after drying at 105°C, and particle density (ρ_z_) is the density of soils solid fractions (without pores).

Saturation (θ_ns_) is a soil property where capillary pores are filled with water (by capillary rise). In the laboratory, it can be used to control the porosity calculations. In non-swelling soils, it is usually lower than porosity. In soils which swell, and the saturation value is greater than porosity, the saturation value can be used instead of porosity.

30-minute moisture (θ_30_) is a soil property used for classification of soil pores. It was established that after 15 minutes (in light) and 30 minutes (in medium-heavy and heavy soils) there is a rapid decrease in moisture from non-capillary pores, due to gravity.

Maximum capillary capacity (θ_MKK_), according to V. Novák, represents the ability of soil to hold plant-accessible water. Experiments of reaching the capillary equilibrium state have proven that at this point, the equilibrium is not yet reached, and the water is still under the influence of gravity. It can be used as the value for soil water retention in cases where, due to time limitations, obtaining the results of water retention capacity (θ_RVK_) is not possible. θ_MKK_ should not be greater than 75-80% porosity. Both extremely high and extremely low values indicate a poor physical state of soil (due to a low amount of non-capillary pores). This usually happens in compacted and poorly structured soils [10]. According to Pospíšilová et al. [8], in clayey soils, θ_MKK_ should not exceed 36%, and if it does, the soil is considered disturbed and water infiltration is poor.

Assessment of soil's water holding properties based on θ_MKK_, according to Rejšek [9] is given in Table 2.

**Table 2:** Water holding properties based on θ_MKK_[9].

Maximum capillary capacity (θ_MKK_) [%]	Water holding properties
< 5	Very poor water retention
5 - 10	Poor water retention
10 - 30	Good water retention
30 - 50	Strong water retention
> 50	Very strong water retention

Water retention capacity (θ_RVK_), unlike the previous one, shows a state of equilibrium between non-capillary and capillary pores close to the theoretical one. Water in the soil is no longer drained by gravity, but exclusively held by capillary forces.

Soil air characteristics go hand in hand with water, and together they make soil porosity.

Aeration (V_Z_) represents the share of pores filled by air at the actual moisture level. It is expressed as a total volume percentage or percentage relative to porosity.

Air capacity is the percentage of pores filled by air at maximum capillary capacity or water retention capacity.

Particle (specific) density (ρ_z_) is the density of the solid fraction of the soil (without pores). The value depends on different particle densities of minerals that are incorporated into the soil sample, and can also be an indirect indicator of the amount of organic matter in the soil (Table 3).

**Table 3:** Assessment of soil organic matter content based on particle (specific) density [15].

Particle density [g/cm^3^]	Assessment
<2.0	peat horizons
2.0-2.4	bog horizons
2.4-2.5	highly humified
2.5-2.6	lightly humified
2.6-2.7	clayey horizons with cca. 1% humus
2.7-2.8	iron-containing illuvial horizons

Porosity is a soil property that defines which amount of the total sample volume, in undisturbed conditions, is made of pores. It is calculated through bulk and particle density. Guidelines for assessing soil porosity are given in Table 4.

**Table 4:** Assessment of soil compaction based on porosity values (in % vol.) [15].

Assessment	Topsoil porosity [%]		Subsoil porosity [%]	
	Light soil	Medium/heavy soil	Light soil	Medium / heavy soil
loose	> 65	> 65	> 50	> 57
moderately compacted	50 - 65	55 - 65	43 - 50	46 - 57
compacted	40 - 50	45 - 55	35 - 43	35 - 46
very compacted	< 40	< 45	< 35	< 35

Pores in the soil can be divided into three categories: capillary, semi-capillary and non-capillary. Capillary pores are numerically equal to θ_RVK_. Semi-capillary are the pores in between capillary and non-capillary, and non-capillary pores are the pores from which water can exit (almost immediately) due to gravity.

An optimal share of capillary pores should be around 2/3 porosity. The rest should be approximately equal portions of semi-capillary and non-capillary pores.

An excessive share of capillary pores makes poor infiltration properties (only small amounts of water reach the profile, water gets infiltrated only in the upper parts of the horizon, excessive runoff and erosion are present, especially on slopes). These soils dry up fast after getting wet. When saturated with water, they have low aeration. Insufficient amounts of capillary pores mean low amounts of plant-accessible water.

Semi-capillary pores provide good infiltration, and the temporary stay of water in them provides saturation of capillary pores with water up to a greater depth.

Non-capillary pores provide water infiltration to the profile and its penetration to deeper layers. Having an excessive share of these pores does not make the upper parts of the profile wet, and the infiltration speed is so great that the water leaks into the deeper layers before being able to saturate the capillary pores. Water supply of these soils is poor.

## Ethics statements

Human subjects, animal testing and social media data collection were not utilized in creating this methodology.

## CRediT authorship contribution statement

**Marko Spasić:** Methodology, Writing – original draft, Visualization. **Oldřich Vacek:** . **Kateřina Vejvodová:** Writing – review & editing, Visualization. **Václav Tejnecký:** Resources, Writing – original draft. **Filip Polák:** Writing – original draft. **Luboš Borůvka:** Funding acquisition. **Ondřej Drábek:** Conceptualization, Supervision.

## Declaration of Competing Interest

The authors declare that they have no known competing financial interests or personal relationships that could have appeared to influence the work reported in this paper.

## Data Availability

No data was used for the research described in the article.
